# Sex- and Female Age-Dependent Differences in Gene Expression in Diffuse Large B-Cell Lymphoma—Possible Estrogen Effects

**DOI:** 10.3390/cancers15041298

**Published:** 2023-02-17

**Authors:** Dan Huang, Mattias Berglund, Anastasios Damdimopoulos, Per Antonson, Cecilia Lindskog, Gunilla Enblad, Rose-Marie Amini, Sam Okret

**Affiliations:** 1 Department of Biosciences and Nutrition, Karolinska Institutet, SE-141 83 Huddinge, Sweden; 2 Bioinformatics and Expression Core Facility, Department of Biosciences and Nutrition, Karolinska Institutet, SE-141 83 Huddinge, Sweden; 3 Department of Immunology, Genetics and Pathology, Uppsala University, SE-751 85 Uppsala, Sweden

**Keywords:** diffuse large B-cell lymphoma, gene expression, RNA sequencing, sex difference, estrogen, pre- vs. postmenopaus, NR4A, MUC5B

## Abstract

**Simple Summary:**

Females show a favorable sex difference in incidence as well as in survival for many cancer types, so also in lymphoma. The reasons for this are unknown. We have therefore analyzed global gene expression in a large cohort of the most common lymphoma type, diffuse large B-cell lymphoma. We show that many genes are differentially expressed between males and females. Furthermore, the results demonstrate sex-dependent differences in gene expression between DLBCL subtypes. In addition, gene expression differs in pre- vs. postmenopausal women suggesting that estrogen regulation of genes is involved. Thus, estrogens may contribute to the sex and female age differences in incidence and prognosis observed.

**Abstract:**

For most lymphomas, including diffuse large B-cell lymphoma (DLBCL), the male incidence is higher, and the prognosis is worse compared to females. The reasons are unclear; however, epidemiological and experimental data suggest that estrogens are involved. With this in mind, we analyzed gene expression data from a publicly available cohort (EGAD00001003600) of 746 DLBCL samples based on RNA sequencing. We found 1293 genes to be differentially expressed between males and females (adj. *p*-value < 0.05). Few autosomal genes and pathways showed common sex-regulated expression between germinal center B-cell (GCB) and activated B-cell lymphoma (ABC) DLBCL. Analysis of differentially expressed genes between pre- vs. postmenopausal females identified 208 GCB and 345 ABC genes, with only 5 being shared. When combining the differentially expressed genes between females vs. males and pre- vs. postmenopausal females, nine putative estrogen-regulated genes were identified in ABC DLBCL. Two of them, *NR4A2* and *MUC5B*, showed induced and repressed expression, respectively. Interestingly, NR4A2 has been reported as a tumor suppressor in lymphoma. We show that ABC DLBCL females with a high *NR4A2* expression showed better survival. Inversely, MUC5B expression causes a more malignant phenotype in several cancers. *NR4A2* and *MUC5B* were confirmed to be estrogen-regulated when the ABC cell line U2932 was grafted to mice. The results demonstrate sex- and female reproductive age-dependent differences in gene expression between DLBCL subtypes, likely due to estrogens. This may contribute to the sex differences in incidence and prognosis.

## 1. Introduction

Diffuse large B-cell lymphoma (DLBCL) is an aggressive lymphoid malignancy and the most common type of B-cell Non-Hodgkin lymphoma (NHL), accounting for about 30% of all lymphomas [1]. Although current first-line therapy with immunochemotherapy regimes cures about 60% of patients, up to 40% of patients still experience relapse or incomplete remission, which represents a therapeutic challenge [2]. To address this aspect, the molecular characterization of DLBCL has been intensively studied. This has made it clear that DLBCL is not a single disease. Gene expression profiling has identified two major subtypes with distinct gene expression patterns, one resembling normal germinal center B cells (denoted GCB) and the other more similar to activated B cells (denoted ABC) [3]. Based on this subdivision, ABC DLBCL patients have, on average, a worse prognosis compared to GCB DLBCL [4]. A few cases that cannot be attributed to one of these two classes are defined as unclassified (UC) [5].

Many studies show a clear sex difference for most types of lymphoma with, in general, higher incidence and poorer prognosis in males compared to females [6,7,8]. In addition, the sex ratio (males vs. females) for several types of lymphoma also varies by age. As an example, for DLBCL, a recent study showed that during young age (15–45 years), DLBCL is about 2–5 times more common in males, while at higher age (>65 years), the difference in incidence is almost eradicated [9]. Likewise, in a population-based cohort, it was found that premenopausal females (younger than 52 years) but not females older than 52 years have better overall survival in DLBCL compared to men of the same age [10].

Furthermore, a decreased incidence with an increased number of pregnancies has been demonstrated [11,12], and in some but not all studies, a lower lymphoma incidence has been correlated to the use of hormone replacement therapy [11,12,13]. Although lymphomas are not considered to be hormone-dependent malignancies, this sex- and female age-dependent variation in sex ratio incidence and prognosis may be due to hormonal differences, particularly of estrogens. In support of this, we have observed faster lymphoma growth in male vs. female mice of xenografted human DLBCL or murine T cell lymphoma [14,15]. Furthermore, when grafting mice with the murine T cell lymphoma, the sex difference in tumor progression disappeared in female mice following ovariectomy, indicating a role for estrogens in suppressing lymphoma growth [15]. In addition, we have shown that the treatment of mice with aromatase inhibitors that prevent estrogen biosynthesis enhances both B and T cell lymphoma growth [16]. In contrast, treating grafted DLBCL cells or other human B-cell lymphoma cells (mantle cell lymphoma and Burkitt lymphoma) with selective estrogen receptor agonists targeting the estrogen receptor β (ERβ/ESR2), the major nuclear estrogen receptor expressed in lymphoma cells [17], impaired tumor progression, reduced dissemination and vascularization, particularly lymphangiogenesis [18]. Interestingly, the selective estrogen receptor modulator tamoxifen has also been shown to reduce lymphoma growth and incidence [19].

Although epidemiological and experimental data suggest a sex difference, possibly mediated by estrogens, for lymphoma incidence and progression, very little is known whether this also is reflected in sex- and age-dependent differences in gene expression and pathway signaling in the DLBCL. To study this, as well as to identify estrogen-regulated genes in DLBCL, we have used gene expression data from a large publically available dataset of clinical DLBCL samples using 746 specimens analyzed by RNA-seq [3]. Some of the genes identified to be potentially regulated by estrogens according to the RNA-seq data were verified to be estrogen-regulated in xenograft experiments and identified to be expressed in tumor cells as analyzed in DLBCL tissue microarray by immunohistochemistry (IHC).

## 2. Materials and Methods

### 2.1. Analysis of RNA Sequencing Data

The bam files of EGAD00001003600 containing the RNA-seq data from the 775 DLBCL patients were downloaded from European Genome-Phenome Archive (EGA, https://www.ega-archive.org/, accessed on 1 November 2020) with permission from the S. Dave lab as described [3] using the pyEGA3 download client (https://ega-archive.org/download/downloader-quickguide-APIv3). RNA-seq data from 29 samples described in the original data set were excluded from our analyses due to insufficient depth of reads or low coverage of genes. Classification of the DLBCL subtypes ABC, GCB, and UC, respectively, was as defined by the S. Dave lab according to the RNA-seq data [3].

Subsequently, featureCounts (v1.5.1) [20] was employed to perform gene counting using the human gene annotation from Ensembl (Homo_sapiens.GRCh37.87.gtf). The read count table from featureCounts was imported into R/Bioconductor. Differential gene expression was performed using DESeq2, which uses the Wald test for significance testing and Cooks’ distance to remove count outliers. It also automatically deletes genes whose mean of normalized counts is below a threshold determined by an optimization procedure [21]. Genes with an adjusted *p*-value < 0.05 were considered statistically significant. R package ClusterProfiler [22] was used for GSEA hallmark pathway analysis. Removal of genes located in chromosomes X and Y was based on Atlas of Genetics and Cytogenetics in Oncology and Haematology (https://atlasgeneticsoncology.org/chromosome-explorer/X/genes, accessed on 1 December 2020 and https://atlasgeneticsoncology.org/chromosome-explorer/Y/genes, accessed on 1 December 2020, respectively).

### 2.2. Immunohistochemistry (IHC) of DLBCL Tissue Microarray (TMA)

Primary DLBCL tissue microarrays (TMAs) were obtained from Uppsala University Hospital [23]. The primary tumor specimens were taken at diagnosis for diagnostic purposes, formalin-fixed, and embedded in paraffin. The TMA and control slides were evaluated independently by two investigators, out of which one is an experienced hematopathologist (R.-M.A.). All tumors included in the TMAs were confirmed to be de novo DLBCL according to the 2008 WHO classification [24]. IHC was performed as described in Supplementary methods.

### 2.3. Cells, Mice, and Grafting Experiments

The human ABC DLBCL cell line U2932 used in this study was obtained from DSMZ (Leibniz Institute, Braunschweig, Germany) and cultured as previously described [14]. Cells were tested free of mycoplasma, and cell authentication was verified by STR-genotyping using the Promega PowerPlex^®^ 21 by Eurofins Genomics (Forensic Department, Ebersberg, Germany).

Immunocompromised non-obese diabetic severe combined immunodeficiency NOD/SCID IL2γ^null^ (NOD.Cg-*Prkdc^scid^ Il2rg*^tm1Wjl^/SzJ) mice (referred to as NSG mice) were from the Jackson Laboratory (Bar Harbor, ME, USA) and bred at the Animal Facility of Karolinska University Hospital (Huddinge, Sweden). Mice were kept under specific pathogen-free conditions in 12-hour light-dark cycles and on a soy-free diet during the experimental period with free access to fresh water and food. For the xenograft experiment, male mice (8–10 weeks of age) were injected subcutaneously with 10 × 10^6^ U2932 cells in 100 μL sterile PBS on the right flank. When the tumor size reached around 70 mm^3^, treatment with the aromatase inhibitor letrozole (10 μg/mouse) (Sigma Aldrich, St. Louis, MO, USA, Cat. No. L6545) dissolved in 10% ethanol/90% rapeseed oil or vehicle alone administrated subcutaneously once a day was initiated. The tumor size was measured daily and calculated as 0.5 × length (mm) × width^2^ (mm). Mice were sacrificed after 16 days of treatment. Tumor tissue was extracted and cut into small pieces and kept in RNAlater (Sigma Aldrich, St. Louis, MO, USA, Cat. No. R0901) at −20 °C for further RNA isolation.

### 2.4. RNA Extraction and Reverse Transcription Quantitative Real-Time PCR (RT-qPCR)

RNA extraction and RT-qPCR using KAPA SYBR FAST (Sigma Aldrich, Cat. No KK4605) were performed as previously described [16]. The ∆∆Ct method was used to calculate relative mRNA expression. Human *RPLP0* (*36B4)* was used as the reference gene. Primers used for RT-qPCR are described in Supplementary Table S1.

### 2.5. Statistical Analysis

The statistical test used for the analysis of grafting experiments and RT-qPCR was an unpaired two-tailed *t*-test using GraphPad Prism 9.0 (GraphPad Software, San Diego, CA, USA). Data were presented as mean ± SD. *p*-value < 0.05 was considered as statistically significant and labeled with symbols * *p* < 0.05, ** *p* < 0.01. For statistical analysis of the RNA-seq data, see above under Section 2.1.

### 2.6. Ethical Considerations

IHC analyses on DLBCL samples were performed on coded samples that were obtained in connection to routine sampling only. Ethical approval was granted by ”Regionala etikprövningsnämnden i Uppsala”, Dnr 2014/233, decision 22 December 2014.

All animal experiments were approved by the Swedish Research Animal Ethics Committee (approval no. 14912-2019) and performed according to the guidelines of the Karolinska Institutet.

## 3. Results

### 3.1. Sex Differences in Gene Expression in DLBCL

**Figure 1:**
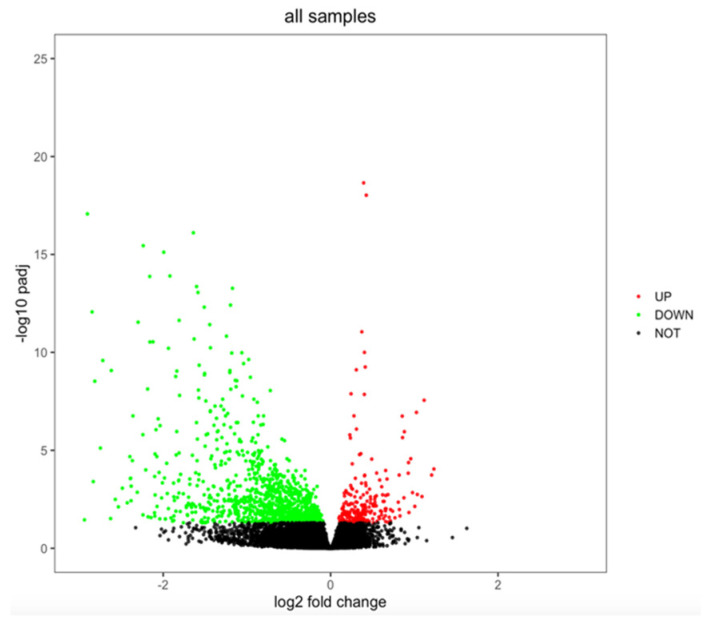
Volcano plot showing sex differences in gene expression in DLBCL. Genes were considered to be differentially expressed when adjusted *p*-value < 0.05. Upregulated genes are marked in red, downregulated genes are marked in green, and not significant genes are marked in black.

When analyzing sex differences in gene expression (females vs. males) among the 746 DLBCL samples to which RNA-seq data, sex, and age information were available (data found in Supplementary Table S2), 1293 genes showed a differential expression (adj. *p*-value < 0.05, Supplementary Table S3). Among the sex-differentiated genes, the majority were downregulated (1075 genes vs. 218 upregulated genes, Figure 1).

**Figure 2:**
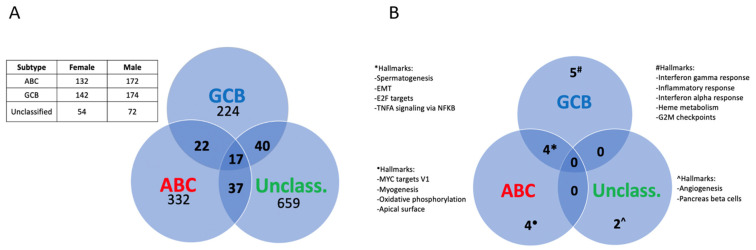
Sex differences in gene expression and pathways among molecular subtypes of DLBCL. (**A**), Venn diagram showing the distribution of the differentially expressed genes among the DLBCL subtypes (adj. *p*-value < 0.05). The table shows the number of samples for each subtype and sex. (**B**), Venn diagram depicting the significant pathways showing a sex difference among the DLBCL subtypes as analyzed by GSEA (adj. *p*-value < 0.05).

In order to look more in depth for possible sex differences among the two major biologically distinct molecular subtypes of DLBCL, GCB, and ABC, as well for DLBCL specimens that cannot be classified as one of the above subtypes (defined as UC [3]), we analyzed sex differences in gene expression in these subtypes separately. As can be seen from Figure 2A, few of the differentially expressed genes were shared and showed the same direction of regulation among the DLBCL subgroups, suggesting a sex difference in gene expression that varies between the DLBCL subgroups. This overall picture was largely unaffected when genes located on the X and Y chromosomes were excluded (Supplementary Figure S1A).

A gene set enrichment analysis (GSEA), based on ranking all genes according to their fold difference in expression, revealed 15 significant pathways (adj. *p*-value < 0.05) that showed a sex difference between the ABC, GCB subtypes, and the UC DLBCL group. However, only four of these pathways were shared between the GCB and ABC DLBCL subtypes, while four were unique for the ABC and five for the GCB subtype, respectively (Figure 2B, Supplementary Tables S4 and S5). These results indicate a sex-dependent difference in signaling pathways between the GCB and ABC DLBCL subtypes. No pathway was common between GCB or ABC with UC DLBCL (Figure 2B). The GSEA analysis was largely unaffected when excluding genes that were localized to the X and Y chromosomes, except that one additional pathway (G2M checkpoint) became similarly regulated in a sex-dependent manner between females vs. males in the GCB and ABC DLBCL subtypes. Furthermore, the GSEA pathway “coagulation” became significant in the ABC subtype and “apoptosis” in the GCB subtype but not overlapping with the sex-differentiated pathways between the subtypes (Supplementary Figure S1B). This excluded sex chromosome genes as a major cause for the differential expression of signaling pathways between the different subtypes in males vs. females. For further studies, we restricted our analysis to the GCB and ABC DLBCL subtypes since the number of UC DLBCL samples was relatively few.

### 3.2. Differences in Gene Expression in DLBCL between Females in Pre- and Postmenopausal Age

The above results, as well as previous results (see Introduction), may suggest that estrogens are responsible for the sex-dependent differences in gene expression observed. In order to support this hypothesis, we compared gene expression in young females ≤ 52 years of age (premenopausal) with females ≥ 65 years of age (postmenopausal) in the GCB and ABC DLBCL subtypes, respectively. The average age of menopause in Western countries is around 52 years of age [25]. Analysis of the UC group was excluded since too few females of age ≤ 52 years were present in this group (Supplementary Table S2).

**Figure 3:**
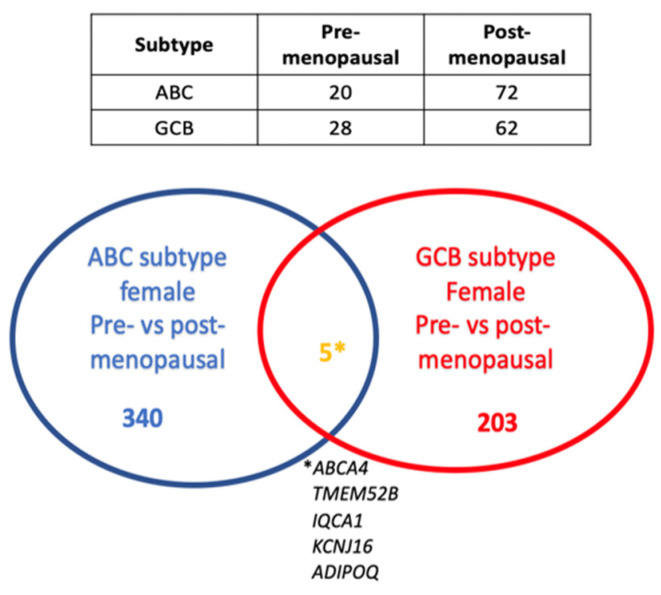
Venn diagram showing the number of genes that are differentially expressed in pre- vs. postmenopausal females among the ABC and GCB subtypes and the overlap of differentially expressed genes in pre- (≤52 years of age) vs. post (≥65 years of age)-menopausal females in the ABC and GCB DLBCL subtypes (adj. *p*-value < 0.05). The table shows the number of DLBCL cases in the ABC and GCB subgroups and in pre- and postmenopausal females, respectively. * This is the explanation (the names) of the five genes that overlap between the GCG and ABC subtypes of female pre- vs. postmenopausal patients.

Analysis of genes differentially expressed in the GCB subtype revealed 208 genes to be differentially expressed in the pre- vs. postmenopausal females, while in the ABC subtype, 345 genes showed a differential expression (adj. *p*-value < 0.05, Supplementary Tables S6 and S7). Interestingly, only five genes with an adj. *p*-value < 0.05 were common between the GCB and ABC subtypes, suggesting that putative estrogen-regulated genes are largely different in GCB and ABC DLBCL (Figure 3). This was substantiated by the fact that no signaling pathway was found to be common between the GCB and ABC subtypes in pre- vs. postmenopausal females when analyzed by GSEA when applying an adj. *p*-value < 0.05). 

**Figure 4:**
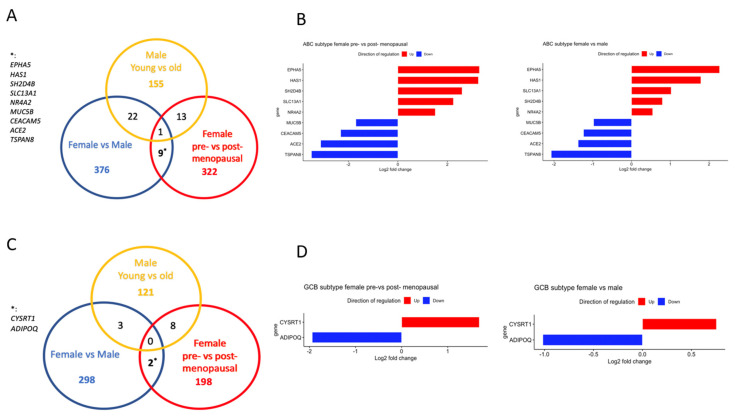
Identification of genes that likely are regulated by estrogens in the ABC and GCB DLBCL subtypes, respectively. (**A**), Venn diagram showing the overlap of differentially expressed genes in the ABC DLBCL subtype. (**B**), Bar graph showing the log2-fold change of genes that are likely regulated by estrogen in the ABC subtype. Upregulated genes are marked in red, and downregulated genes are marked in blue. (**C**), Venn diagram showing the overlap of differentially expressed genes in the GCB DLBCL subtype. (**D**), Bar graph showing the log2-fold change of genes that are likely regulated by estrogens in the GCB subtype. Upregulated genes are marked in red, and downregulated genes are marked in blue.

In order to strengthen the identification of likely estrogen-regulated genes, we identified genes that were altered in the same direction in both pre- vs. postmenopausal females and females vs. males in ABC and GCB DLBCL with an adj. *p*-value < 0.05. Ten genes were found in the ABC subgroup that fulfilled the criteria (Figure 4A,B). However, 1 of the 10 genes (*CAPN8*) was found to be differentially regulated also in young vs. old males and, therefore, most likely represents an age-dependent change in gene expression independent of estrogen influence. In GCB DLBCL, only two genes were identified as potentially being under estrogen control and regulated in the same direction (Figure 4C,D).

### 3.3. Regulation of NR4A and MUC5B Expression in ABC DLBCL by Estrogens

**Figure 5:**
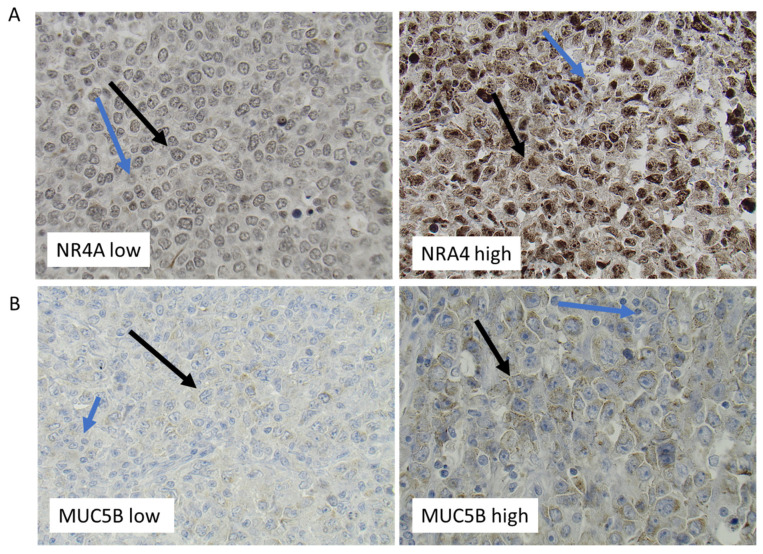
Representative immunohistochemistry staining of DLBCL lymphoma tissue for NR4A2 (**A**) and MUC5B (**B**). Immunohistochemical staining of DLBCL for NR4A2 (**A**) and MUC5B (**B**). Representative staining of samples with low (**left**) and high (**right**) expression, respectively. Black arrows mark examples of expression of NR4A2 and MUC5B in tumor cells, while blue arrows mark expression in non-malignant cells.

Among the nine genes in the ABC subtype that fulfilled the criteria of showing a significant difference in gene expression between females and males and between pre- vs. postmenopausal females, but not being differentially regulated in young vs. old males, were *NR4A2* and *MUC5B* (Figure 4A,B, Supplementary Tables S7 and S8). The *NR4A2* gene was found to be of particular interest since members of the *NR4A* family of genes have been described to act as tumor suppressors in lymphoid malignancies [26]. IHC analysis of DLBCL samples in a TMA showed that NR4A2 protein was expressed in the tumor cells of almost all DLBCL samples with different levels of expression between the samples (Figure 5A). Only 11% of the DLBCL samples showed no or very low NR4A2 expression. 

**Figure 6:**
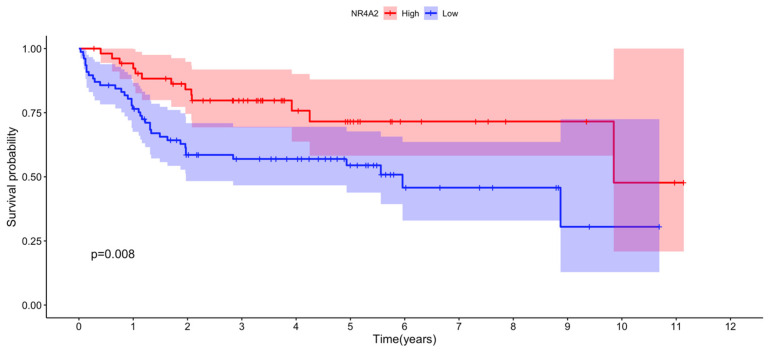
Correlation between *NR4A2* expression and overall survival among female ABC DLBCL patients. *NR4A2* expression data for each individual female ABC DLBCL and individual survival data were obtained from the EGAD00001003600 data set. Optimal cut point for high vs. low *NR4A2* expression was determined using maximally selected rank statistics from the “maxstat” R package. The number of individuals with high and low *NR4A2* expression after optimal cut point determination was 55 and 77, respectively. The R packages “survival” and “survminer” were used for the survival analysis. The pink and blue areas show lower 95% to upper 95% CI for low and high *NR4A2* expression, respectively. *p* = 0.008 for the difference in survival between the high and low *NR4A2*-expressing individuals.

Notably, expression of the *NR4A2* gene in the RNA-seq analysis was significantly upregulated in females vs. males and in pre- vs. postmenopausal females ABC DLBCL, making its expression very likely to be stimulated by estrogens. A closer analysis of the results showed that also the other two members, *NR4A1* and *NR4A3,* were closely fulfilling these criteria. *NR4A1* expression was found to be significantly upregulated when comparing females vs. males (adj. *p*-value = 0.014, Supplementary Table S8) and close to significant for upregulated expression in pre- vs. postmenopausal females (adj. *p*-value = 0.07, Supplementary Table S7). The corresponding adj. *p*-values for stimulated *NR4A3* expression were for pre- vs. postmenopausal females and females vs. males 0.059 and 0.210, respectively (Supplementary Tables S7 and S8). No significance or trend for regulation of *NR4A* family members in GCB DLBCL was observed, neither when comparing pre- vs. postmenopausal women nor in females vs. males (Supplementary Tables S6 and S9). Markedly, female ABC DLBCL cases with high *NR4A2* expression showed a better overall survival compared to cases with low expression when analyzing the EGAD00001003600 data set (*p* = 0.008, Figure 6). No significant correlation between *NR4A1, NR4A2* or *NR4A3* expression and survival was found in female GCB DLBCL.

In contrast to NR4As, which act as tumor suppressors in most lymphoid malignancies, overexpression of mucin genes acts as promoters in several cancer forms [27]. *MUC5B* expression was found in the ABC DLBCL RNA-seq analysis fulfilling the criteria’s of being significantly suppressed in pre- vs. postmenopausal women and females vs. males (Figure 4B and Supplementary Tables S7 and S8) but not showing any differential expression between young vs. old males. This strongly suggests that *MUC5B* expression in ABC DLBCL is suppressed by estrogens. In contrast to the regulation of *MUC5B* expression in ABC DLBCL, as determined by the RNA-seq, no indication of sex- or female reproductive age-dependent regulation of *MUC5B* in GCB DLBCL was observed (Supplementary Tables S6 and S9). IHC analysis verified MUC5B protein expression in a restricted number of DLBCL samples, with these expressing different amounts of MUC5B (Figure 5B).

**Figure 7:**
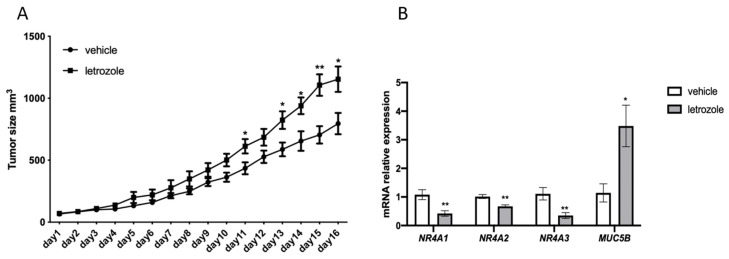
Inhibition of estrogen synthesis by the aromatase inhibitor letrozole stimulates the growth of U2932 tumors and affects the expression of *NR4A* and *MUC5B*. (**A**), Male NSG mice were injected subcutaneously with U2932 cells and treated subcutaneously daily with vehicle or the aromatase inhibitor letrozole (10 μg/mouse). The vehicle group consisted of 7 mice, letrozole group consisted of 8 mice. Tumor size was measured daily. (**B**), RT-qPCR analysis of *NR4A1*, *NR4A2*, *NR4A3* and *MUC5B*. Results are presented as relative expressions (mean ± SD). An unpaired two-tailed t-test was used for statistical analysis between the two groups (* *p* < 0.05, ** *p* < 0.01).

In order to verify estrogen regulation of *NR4A*’s and *MUC5B* in the ABC subgroup of DLBCL in an experimental setting, we grafted the human ABC DLBCL cell line U2932 to immunocompromised mice. Mice were treated with subcutaneous injections of the aromatase inhibitor letrozole, which inhibits endogenous estrogen synthesis [28]. As can be seen from Figure 7A, treatment with letrozole resulted in faster tumor growth as compared to mice treated with vehicle alone. Analysis of *NR4A* gene expression in the tumors at the end of the treatment period showed that expression of *NR4A1, NR4A2,* as well as *NR4A3* were significantly downregulated following letrozole treatment, strongly supporting the estrogen-stimulated expression of these genes (Figure 7B). In contrast, *MUC5B* expression increased following letrozole treatment of the mice suggesting that estrogens exert a suppressive effect on *MUC5B* expression (Figure 7B). The remaining seven genes in the ABC DLBCL identified likely to be under estrogen regulation could not be verified in the U2932 xenograft tumors due to too low expression. Furthermore, their connection to cancer processes is unclear. Likewise, the two genes identified in GCB DLBCL likely to be under estrogen regulation (*ADIPOQ* and *CYSRT1*) were also expressed at very low levels, as seen in the RNA-seq data (Supplementary Table S9).

## 4. Discussion

The sex of a patient is a relevant variable for DLBCL, both with regard to incidence and prognosis. The sex difference in incidence and prognosis is primarily seen in premenopausal women compared to males of the same age interval [9,10], indicating that endocrine factors may play a role. Both epidemiological data and experimental results support a “protective” function of estrogens [6,8,14]. However, molecular information as to how sex and possibly estrogens play a role in NHL in general or in DLBCL specifically is still lacking [29]. Despite this, sex or female reproductive age is not included in the international prognostic index for DLBCL, although several studies have shown that the female sex is a favorable prognostic indicator for overall survival in DLBCL patients treated with rituximab-containing regimens [30]. Notably, though, the favorable outcome for females compared to males has been suggested to be due to women responding better to rituximab [31]. However, also in DLBCL treated with chemotherapy alone (without rituximab), the female sex has a positive impact on overall and progression-free survival compared to males [32]. In addition, the main sex-dependent difference in survival is seen in young females vs. young males in comparison to older females vs. older males [10]. This is further supported by an experiment in mice where tumor progression is slower in fertile female vs. fertile male mice as well as in intact vs. ovariectomized mice not exposed to rituximab [14,15]. This supports that estrogen affects survival independent of rituximab.

In this report, we describe significant sex differences in gene expression in DLBCL. We found 1293 genes that were differentially expressed in the DLBCL samples when comparing females vs. males (adj. *p*-value < 0.05). Furthermore, these differentially expressed genes scarcely overlapped between the ABC, GCB subtypes, and the UC DLBCL group, respectively. This suggests that the sex-mediated effects of gene expression in the DLBCL subtypes are different. This observation was supported by the GSEA, which showed that the sex-dependent signaling pathways observed in the ABC vs. GCB DLBCL subtypes overlapped only partially. The difference toward the UC DLBCL group was even larger. This may reflect that the UC is more different from the ABC and GCB subtypes or that the UC group is more heterogeneous. Importantly though, the sex difference between the DLBCL subtypes was largely unaffected when the sex chromosome genes were excluded from the analysis.

In order to test if the sex- and female age-dependent differences were due to a difference in estrogen levels, we searched for genes that fulfilled the criteria of both being differentially expressed between females and males and between pre- vs. postmenopausal females but not showing an age-dependent difference in expression in males. Using these stringent requirements, one of the genes identified by RNA-seq in the ABC DLBCL subtype was *NR4A2*. NR4A2 protein expression in the tumor cells was confirmed by IHC. However, regulation by sex and female reproductive age could not be confirmed in the available TMA cohort, likely due to too few samples when subdividing according to sex and age.

In addition, the other two *NR4A* family members, *NR4A1* and *NR4A3,* showed a trend of similar regulation, although not fulfilling significant regulation for both criterias (females vs. males and pre- vs postmenopausal females) when analyzing the RNA-seq data. Overall, expression of *NR4A’s* was higher in females vs. males and in pre- vs. postmenopausal women even if not significant, suggesting that expression of these genes indeed is stimulated by estrogens. 

In xenograft experiments using the U2932 ABC DLBCL cell line, which, like DLBCL tumors, express ERβ but no ERα [14], we confirmed estrogen regulation of not only *NR4A2* but also *NR4A1* and *NR4A3*. Inhibition of endogenous estrogen synthesis in the grafted mice using the aromatase inhibitor letrozole reduced the expression of all three *NR4A* family members. To note is that estrogen regulation of *NR4A* gene family members has been described in white adipose tissue and skeletal muscle cells [33,34]. Using the same criteria as above, an indication of estrogen regulation of *NR4A* expression in the GCB DLBCL could not be found, also stressing a difference in estrogen response between the ABC and GCB subtypes. 

While in solid tumor-derived cell lines, *NR4A1* and *NR4A2* expression are high, and both receptors exhibit pro-oncogenic activities, in blood-derived tumors (leukemias and lymphomas), *NR4A* expression, particularly of *NR4A1* and *NR4A3*, is low [26,35]. Although we did not compare *NR4A2* expression in DLBCL compared to non-neoplastic lymphoid tissue, *NR4A2* transcripts, and protein were clearly detected in DLBCL tissue. This was in contrast to a study by Deutsch et al. [35]. The reason for the discrepancy is not clear but may be explained by different technologies used (qPCR vs. RNA sequencing or IHC using antibodies with different sensitivity). Importantly though, a tumor suppressor activity for NR4A’s in blood-derived tumors was shown in *NR4A1^−/−^/NR4A3^−/−^* double knockout mice, which rapidly develop acute myelocytic leukemia [36]. Moreover, overexpression of *NR4A1* or *NR4A3* in lymphoma cells induced apoptosis, supporting a tumor-suppressive function in lymphomas [35,37]. An induction of pro-apoptotic genes and enhancement of apoptosis was also seen when treating leukemia and most lymphoma cells in vitro with drugs that induce the expression of *NR4A1* and *NR4A3* [35,36,37]. Furthermore, a low *NR4A1* expression was demonstrated to be associated with aggressive forms of DLBCL and poor patient survival [35]. In another study, it was described that also a decreased expression of *NR4A3* was associated with poor survival [37]. Similarly, we show that low *NR4A2* expression in ABC DLBCL confers poor survival. Given the above-reported functions of NR4A acting as suppressors of lymphomagenesis and our demonstration of estrogen-induced expression of the *NR4A* members suggests that NR4A in ABC DLBCL contribute to the sex- and female reproductive age-dependent differences in lymphoma incidence and prognosis. An effect of estrogens on lymphoma growth is further supported by our previous experimental observations that the administration of estrogens, particularly by ERβ agonists, inhibits DLBCL progression or that ovariectomy of mice abolished sex difference of lymphoma growth [14,15]. Of interest is also an observation in our lab that ERβ interacts with the promoter region of *NR4A2* in the genome of the mantle cell lymphoma cell line Granta-519, as determined by genome-wide chromatin immunoprecipitation [38]. This suggests that *NR4A2* is a direct target of ERβ.

One of the other genes identified in the ABC DLBCL subtype being under estrogen control, confirmed by RT-qPCR in the U2932 ABC cell grafting experiment, was the mucin gene *MUC5B*. Inhibition of estrogen synthesis was found to increase *MUC5B* expression. Notably, increased MUC5B expression is associated with increased aggressive behavior of breast cancer and colorectal cancer [39,40]. Moreover, in non-small cell lung cancer, MUC5B expression is significantly associated with poorer differentiation, pathological stage, and poor prognosis [41]. In vitro, down-regulation of MUC5B in gastric cancer and colonic cancer cells leads to a decrease in proliferation, migration, and invasion properties. In addition, in vivo xenografts of MUC5B-deficient cells resulted in a decrease in tumor growth when compared with xenografts of MUC5B-expressing mock cells [42]. However, a role for dysregulated *MUC5B* expression in DLBCL has not been described, although mutations of the *MUC5B* gene have been reported in DLBCL stromal cells [43]. While the RNA-seq data cannot distinguish between the tumor cell and stromal cell expression of *MUC5B* or *NR4A*, the use of highly human-specific primers when analyzing the expression of these genes in human U2932 tumor xenografts by RT-qPCR supports that the expression occurs in the tumor cells. NR4A2 and MUC5B protein expression in tumor cells was confirmed by IHC analysis of DLBCL samples (Figure 5A,B). However, this does not exclude concomitant expression in cells of the tumor microenvironment. In fact, our IHC analysis of DLBCL samples also showed expression in the tumor microenvironment (Figure 5A,B). Given that a decreased expression of MUC5B is associated with a less malignant phenotype in several cancers and that estrogens down-regulate *MUC5B* expression in ABC DLBCL, it is not unlikely that estrogens by reducing *MUC5B* expression contribute (with or without an increase in *NR4A* expression) to a reduction in ABC DLBCL progression. Additionally, a report has shown that 17β-estradiol regulates *MUC5B* expression in airway epithelial cells, although in the opposite direction compared to our observation in DLBCL, supporting that *MUC5B* indeed is an estrogen-regulated gene [44]. The opposite direction of *MUC5B* gene expression by estrogens in different tissues may, e.g., be dependent on cell-specific differences in the expression of estrogen receptor isoforms or signaling pathways involved [45,46]. No regulation by estrogens of *MUC5B* as well as *NR4A* expression, was identified in GCB DLBCL. This suggests that the mechanisms by which estrogens affect ABC vs. GCB DLBCL are different, further strengthening that the sex effect on these subtypes is distinct. Since NR4A and MUC5B are not only expressed in the DLBCL cells but also in non-malignant cells, we cannot exclude that estrogen effects on expression in non-malignant cells, e.g., cells of the immune system, contribute to the response [47,48,49,50]. However, it should be noted that estrogen suppression of DLBCL growth still occurs in immunocompromised mice lacking T, B, and NK cells, excluding a role for lymphoid cells in the response [14].

Notably, our study does not exclude other genes than the ones described above that contribute to the sex- and female reproductive age-specific differences, especially since we identified putative estrogen-regulated genes using stringent criteria. Furthermore, confirmation of estrogen regulation of identified genes on the protein level in larger cohorts of clinical DLBCL samples would strengthen the conclusion.

## 5. Conclusions

In this report, we demonstrate sex-dependent differences in gene expression in DLBCL that differ considerably between the ABC and the GCB DLBCL subtypes. We also demonstrate an age-dependent difference in gene expression in females when comparing women in pre- vs. postmenopausal age. The differences are likely due to estrogen effects. We identify the *NR4A* and *MUC5B* genes in ABC DLBCL being under estrogen control, with the tumor-suppressing *NR4A* gene family members being induced and the tumor-stimulating *MUC5B* being repressed. Given the DLBCL tumor-stimulating effects by inhibition of estrogen synthesis in xenograft experiments, it is likely that the suppressor function of NR4A and the pro-oncogenic activity of MUC5B contribute to a reduced tumor growth of ABC DLBCL. The results demonstrate that DLBCL is under endocrine control by estrogens. However, the response to estrogens differs between the ABC and GCB DLBCL. The estrogen effects may contribute to the understanding of sex-dependent differences in DLBCL incidence and prognosis. Furthermore, new treatment options targeting estrogen signaling by selective ERβ agonists, thereby avoiding ERα effects, or by treatment with estrogen receptor modulators such as tamoxifen, which acts as an ERβ agonist on DLBCL, may be applied in the future [19].

## Data Availability

Additional information is also available in Supplementary Tables and Figures.

## Supplementary Materials

The following supporting information can be downloaded at: https://www.mdpi.com/article/10.3390/cancers15041298/s1, Figure S1A,B: Sex differences in gene expression and pathways among molecular subtypes of DLBCL after removing genes located on X and Y chromosomes; Figure S1: Representative immunohistochemistry staining of ABC DLBCL lymphoma tissue for NR4A2 (A) and MUC5B (B). Table S1: Primers used in RT-qPCR; Table S2: Clinical information of DLBCL cohort; Table S3: Differential gene expression in females vs. males; Table S4: GCB DLBCL subtype, female vs. male hallmark pathway analysis; Table S5: ABC DLBCL subtype, female vs. male hallmark pathway analysis; Table S6: GCB DLBCL subtype, genes differentially in pre- vs. postmenopausal females; Table S7: ABC DLBCL subtype, genes differentially in pre- vs. postmenopausal females; Table S8: ABC DLBCL subtype, genes differentially expressed females vs. males; Table S9: GCB DLBCL subtype, genes differentially expressed females vs. males. Supplementary methods: Immunohostochemistry.
