# Supplementary material for: Sex- and Female Age-Dependent Differences in Gene Expression in Diffuse Large B-Cell Lymphoma—Possible Estrogen Effects
Source: Cancers (Basel). 2023 Feb 17;15(4):1298. doi: 10.3390/cancers15041298 (PMC9954534; doi:10.3390/cancers15041298)
Supplement: Supplementary file 1 [file cancers-15-01298-s001.zip › cancers-2141934-supplementary/Suppl Figures with legends.pdf]

Supplementary Figures

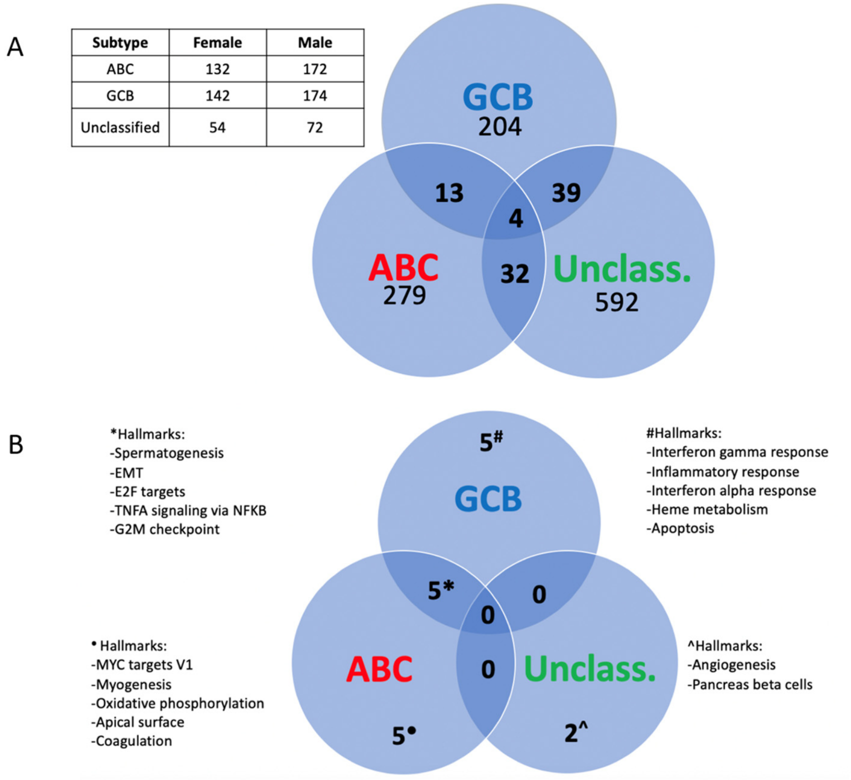

Subtype	Female	Male
ABC	132	172
GCB	142	174
Unclassified	54	72

Supplementary Figure S1: Sex differences in gene expression and pathways among molecular subtypes of DLBCL after removing genes located on X and Y chromosome. A, Venn diagram showing the distribution of the differentially expressed genes among the DLBCL subtypes (adj. p-value <0.05). B, Venn diagram depicting the significant pathways showing a sex differentiation among the DLBCL subtypes as analyzed by GSEA (adj. p-value <0.05).
