# Supplementary material for: Sex- and Female Age-Dependent Differences in Gene Expression in Diffuse Large B-Cell Lymphoma—Possible Estrogen Effects
Source: Cancers (Basel). 2023 Feb 17;15(4):1298. doi: 10.3390/cancers15041298 (PMC9954534; doi:10.3390/cancers15041298)
Supplement: Supplementary file 1 [file cancers-15-01298-s001.zip › cancers-2141934-supplementary/Suppl. Table S1 primers for RT-qPCR.pdf]

Supplementary Table S1.

Primers for RT-qPCR

	**Forward Sequence (5'-3')**	**Reverse Sequence (5'-3')**
NR4A1	ACAGAAGAACTTCGGGAGCG	CGGTGCTGGTGTCCCATATT
NR4A2	CGTGAGGTGTCCGGGTTC	CAGCCTGTCCAATCTCCTCC
NR4A3	AGCCGCTGGGCTTGTA	TGCCCTCTTCCGCAGTG
MUC5B	GTGACAACCGTGTCGTCCTG	TGCCGTCAAAGGTGGAATAG
RPLP0	TTCTCGCTTCCTGGAGGGTG	GACAAGGCCAGGACTCGTTT
