# Supplementary material for: Sex- and Female Age-Dependent Differences in Gene Expression in Diffuse Large B-Cell Lymphoma—Possible Estrogen Effects
Source: Cancers (Basel). 2023 Feb 17;15(4):1298. doi: 10.3390/cancers15041298 (PMC9954534; doi:10.3390/cancers15041298)
Supplement: Supplementary file 1 [file cancers-15-01298-s001.zip › cancers-2141934-supplementary/Suppl. Table S2 Clinical information of the DLBCL cohort.pdf]

**Supplementary Table S2. Clinical information of DLBCL cohort.**

| ABC Subtype | | | | | |
|---|---|---|---|---|---|
| Gender | Young(age<=52) | Middle | Old(age>=65) | NA | Total |
| Female | 20 | 32 | 72 | 8 | 132 |
| Male | 38 | 54 | 76 | 4 | 172 |
| Total | 58 | 86 | 148 | 12 | 304 |

| Unclassified Subtype | | | | | |
|---|---|---|---|---|---|
| Gender | Young(age<=52) | Middle | Old(age>=65) | NA | Total |
| Female | 10 | 13 | 26 | 5 | 54 |
| Male | 24 | 15 | 32 | 1 | 72 |
| Total | 34 | 28 | 58 | 6 | 126 |

| GCB Subtype | | | | | |
|---|---|---|---|---|---|
| Gender | Young(age<=52) | Middle | Old(age>=65) | NA | Total |
| Female | 28 | 44 | 62 | 8 | 142 |
| Male | 57 | 54 | 58 | 5 | 174 |
| Total | 85 | 98 | 120 | 13 | 316 |
