# Supplementary material for: Sex- and Female Age-Dependent Differences in Gene Expression in Diffuse Large B-Cell Lymphoma—Possible Estrogen Effects
Source: Cancers (Basel). 2023 Feb 17;15(4):1298. doi: 10.3390/cancers15041298 (PMC9954534; doi:10.3390/cancers15041298)
Supplement: Supplementary file 1 [file cancers-15-01298-s001.zip › cancers-2141934-supplementary/Supplementary methods.pdf]

Supplementary methods

Immunohostochemistry

TMA slides were stained for NR4A2 and MUC5B using a standard immunohistochemical (IHC) protocol from the Human Protein Atlas (https://www.proteinatlas.org). In brief, tissue slides were deparaffinized and hydrated in xylene and graded ethanol, respectively. Endogenous peroxidases were blocked in 0.3% hydrogen peroxide in 95% ethanol, for 5 min. For antigen retrieval, slides were boiled in Dako Target Retrieval Solution (Agilent Technologies, Santa Clara, CA, USA), pH 6, at 125°C for 4 min in a pressure boiler (Decloaking chamber, Biocare Medical, Walnut Creek, CA, USA) followed by cooling to 90°C, in total a protocol of ∼40 min. Automated IHC staining was performed using Autostainer 480 (ThermoFisher Scientific, Waltham, MA, USA). Slides were blocked with Ultra V Block for 5 min, followed by washing and incubation with primary antibodies for 30 min, NR4A2 antibody (cat. no. 10975-2-AP, obtained from Proteintech, Manchester, United Kingdom at a dilution of 1/250) and MUC5B (cat. no. sc-20119 obtained from Santa Cruz Biotechnology, Dallas, USA at a dilution of 1/100). Next, slides were rinsed in wash buffer and incubated for 30 min with UltraVision LP HRP Polymer visualization probe (ThermoFisher Scientific) for 30 min and developed with Diaminobenzidine (ThermoFisher Scientific) for 10 min. Slides were then counterstained with Mayer's hematoxylin (Histolab Products AB, Göteborg, Sweden) and mounted with Pertex mounting medium. Normal human bone marrow served as positive control for NR4A2 staining while salivary gland served as positive control for MUC5B staining. Human normal skin and omission of the primary antibody served as negative controls for both antibodies. Intensity in positive tumor cells was determined at 400x magnification. Each tumor sample was given a value for expression between 0 (negative) to 5 (high).
